# Treating (low-risk) DCIS patients: What can we learn from real-world cancer registry evidence?

**DOI:** 10.1007/s10549-020-06042-1

**Published:** 2021-01-03

**Authors:** Danalyn Byng, Valesca P. Retèl, Michael Schaapveld, Jelle Wesseling, Wim H. van Harten, on behalf of the Grand Challenge PRECISION consortium

**Affiliations:** 1 Division of Psychosocial Research and Epidemiology, The Netherlands Cancer Institute-Antoni van Leeuwenhoek Hospital, Plesmanlaan 121, 1066 CX Amsterdam, The Netherlands; 2 Health Technology and Services Research Department, Technical Medical Centre, University of Twente, P.O. Box 217, 7500 AE Enschede, The Netherlands; 3 Division of Molecular Pathology, The Netherlands Cancer Institute-Antoni van Leeuwenhoek Hospital, Plesmanlaan 121, 1066 CX Amsterdam, The Netherlands

**Keywords:** Ductal carcinoma in situ, Active surveillance, Multi-state modeling, Real-world data

## Abstract

**Purpose:**

Results from active surveillance trials for ductal carcinoma in situ (DCIS) will not be available for > 10 years. A model based on real-world data (RWD) can demonstrate the comparative impact of non-intervention for women with low-risk features.

**Methods:**

Multi-state models were developed using Surveillance, Epidemiology, and End Results Program (SEER) data for three treatment strategies (no local treatment, breast conserving surgery [BCS], BCS + radiotherapy [RT]), and for women with DCIS low-risk features. Eligible cases included women aged ≥ 40 years, diagnosed with primary DCIS between 1992 and 2016. Five mutually exclusive health states were modelled: DCIS, ipsilateral invasive breast cancer (iIBC) ≤ 5 years and > 5 years post-DCIS diagnosis, contralateral IBC, death preceded by and death not preceded by IBC. Propensity score-weighted Cox models assessed effects of treatment, age, diagnosis year, grade, ER status, and race.

**Results:**

Data on *n* = 85,982 women were used. Increased risk of iIBC ≤ 5 years post-DCIS was demonstrated for ages 40–49 (Hazard ratio (HR) 1.86, 95% Confidence Interval (CI) 1.34–2.57 compared to age 50–69), grade 3 lesions (HR 1.42, 95%CI 1.05-1.91) compared to grade 2, lesion size ≥ 2 cm (HR 1.66, 95%CI 1.23–2.25), and Black race (HR 2.52, 95%CI 1.83–3.48 compared to White). According to the multi-state model, propensity score-matched women with low-risk features who had not died or experienced any subsequent breast event by 10 years, had a predicted probability of iIBC as first event of 3.02% for no local treatment, 1.66% for BCS, and 0.42% for BCS+RT.

**Conclusion:**

RWD from the SEER registry showed that women with primary DCIS and low-risk features demonstrate minimal differences by treatment strategy in experiencing subsequent breast events. There may be opportunity to de-escalate treatment for certain women with low-risk features: Hispanic and non-Hispanic white women aged 50–69 at diagnosis, with ER+, grade 1 + 2, < 2 cm DCIS lesions.

**Supplementary Information:**

The online version of this article (10.1007/s10549-020-06042-1) contains supplementary material, which is available to authorized users.

## Introduction

Women with asymptomatic ductal carcinoma in situ (DCIS) represent a growing proportion of women diagnosed through breast cancer screening programs [1, 2]. Localized treatment strategies for DCIS demonstrate no direct survival benefit to patients [3, 4]. Surgical removal of the lesion, possibly followed by radiation, is intended to lessen the risk of a subsequent ipsilateral invasive breast cancer (iIBC) and its associated mortality risk. Treatment-related adverse events following surgery and radiotherapy have a profound impact on quality of life over the first 24 months following treatment and there is concern that the active treatment of DCIS represents significant overtreatment for some individuals who will never develop invasive disease within their lifetime [5].

As all DCIS lesions are treated, the natural disease course of DCIS remains unclear: estimates show a range of 14–53% of untreated DCIS progressing to invasive cancer over a period of 10 or more years [6]. This is a heterogeneous disease, with certain clinicopathologic characteristics known to be highly prognostic of iIBC after DCIS diagnosis, such as premenopausal status, detection by palpation, involved margins, high histologic grade, and high p16 expression [7]. Studies are ongoing to understand risk of progression from DCIS from a genomic perspective [8]. For women with a combination of low-risk clinicopathological features within the DCIS population, the risk of subsequent iIBC has not yet been quantified. Now, discussions surrounding the safe de-escalation of treatment of DCIS have taken center-stage to address this knowledge gap. An active surveillance strategy has been proposed for patients with low-risk prognostic features, including low-grade and smaller, estrogen receptor positive (ER+) lesions. This allows for the prioritization of a woman’s quality of life: acknowledging that preventing breast cancer is not merely a question of tackling risk factors, but upholding the value of a life minimally affected by treatment-related morbidity. The international PRECISION (PREvent ductal Carcinoma In Situ Invasive Overtreatment Now) initiative is overseeing three clinical trials of active surveillance for low risk DCIS: Comparison of Operative to Monitoring and Endocrine Therapy (COMET), Low Risk DCIS (LORD) and Low RISk DCIS (LORIS) [9-11]. These trials compare safety and clinical outcomes between patients undergoing standard interventional treatment, and those following an active surveillance strategy with regular mammographic screening.

These studies are on-going, and results will not be available for 10–20 years. Ahead of prospective data from clinical trials, real-world cancer registry data on DCIS can be used to demonstrate how women with low-risk features progress from DCIS to IBC and death. We specifically sought to identify a cohort of women with low-grade, small (< 2 cm), ER+ lesions to who did not receive local-regional treatment to understand the potential impact of an active surveillance strategy compared to standard interventional treatment on health outcomes over a patient’s lifetime. Using real-world cancer registry data from the National Cancer Institute’s Surveillance, Epidemiology, and End Results (SEER) program on locally treated and untreated DCIS patients, we developed a continuous time multi-state Markov model of disease progression for DCIS, integrating patient-level covariates and treatment information. The SEER database records subsequent invasive breast cancer cases after a DCIS diagnosis as new primaries, allowing for the modeling of breast cancer-specific disease progression over a patients’ lifetime.

## Methods

### SEER patient cohort selection

**Fig. 1:**
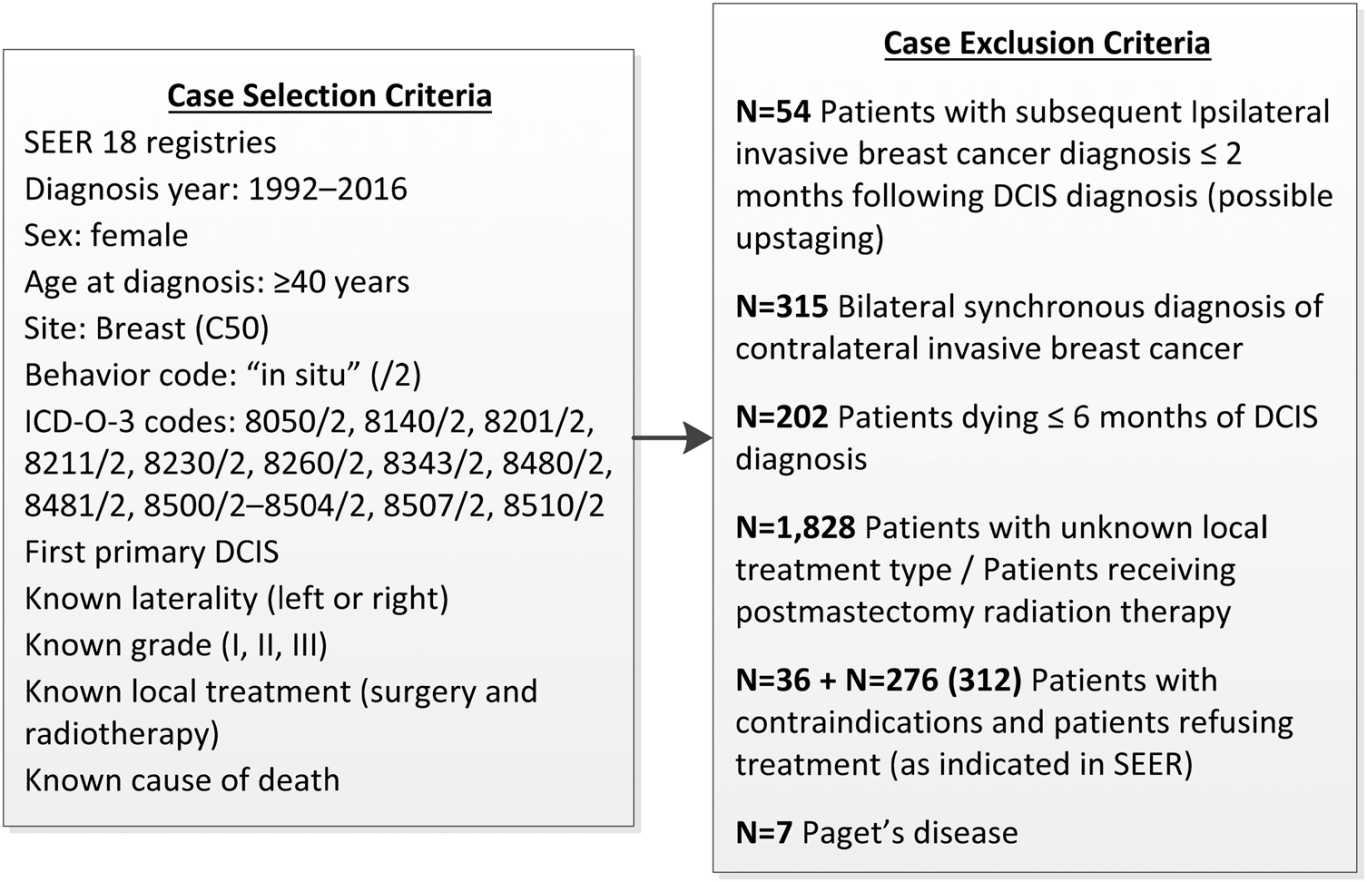
Surveillance, epidemiology, and end results (SEER) case selection and exclusion criteria

Retrospective patient-level data from the SEER 18 registries database (with additional treatment fields on radiation therapy) were used for multi-state modeling of disease progression. Eligible cases included women with grade I, II, and III histologically confirmed DCIS as first primary, diagnosed between 1992 and 2016, aged ≥ 40 years at diagnosis, and with known laterality, local treatment status (surgery and radiotherapy), survival time, and cause of death. Exclusion was warranted under any of the following criteria: iIBC ≤ 2 months following DCIS as this might signify upstaging of the DCIS lesion to invasive carcinoma; death of any cause ≤6 months following DCIS diagnosis; synchronous diagnosis of contralateral invasive carcinoma (cIBC); Paget’s disease; patients treated with postmastectomy radiation therapy; and patients not receiving treatment due to comorbidities or refusal (as coded in SEER). Figure 1 shows the numbers of cases excluded*.*

### Capturing local invasive recurrences in SEER

To understand the impact of changes in SEER coding rules in 2007 which may have led to the under-reporting of subsequent iIBC following DCIS, we calculated the annual iIBC incidence density rate in the 5 years pre- and post-2007. This calculation is based on the number of iIBC events in each annual period, divided by the product of the person-time of the at-risk population during each period. This is presented for the full cohort (all risk groups), and by treatment group to account for changing treatment patterns.

### Model building and statistical analysis

**Fig. 2:**
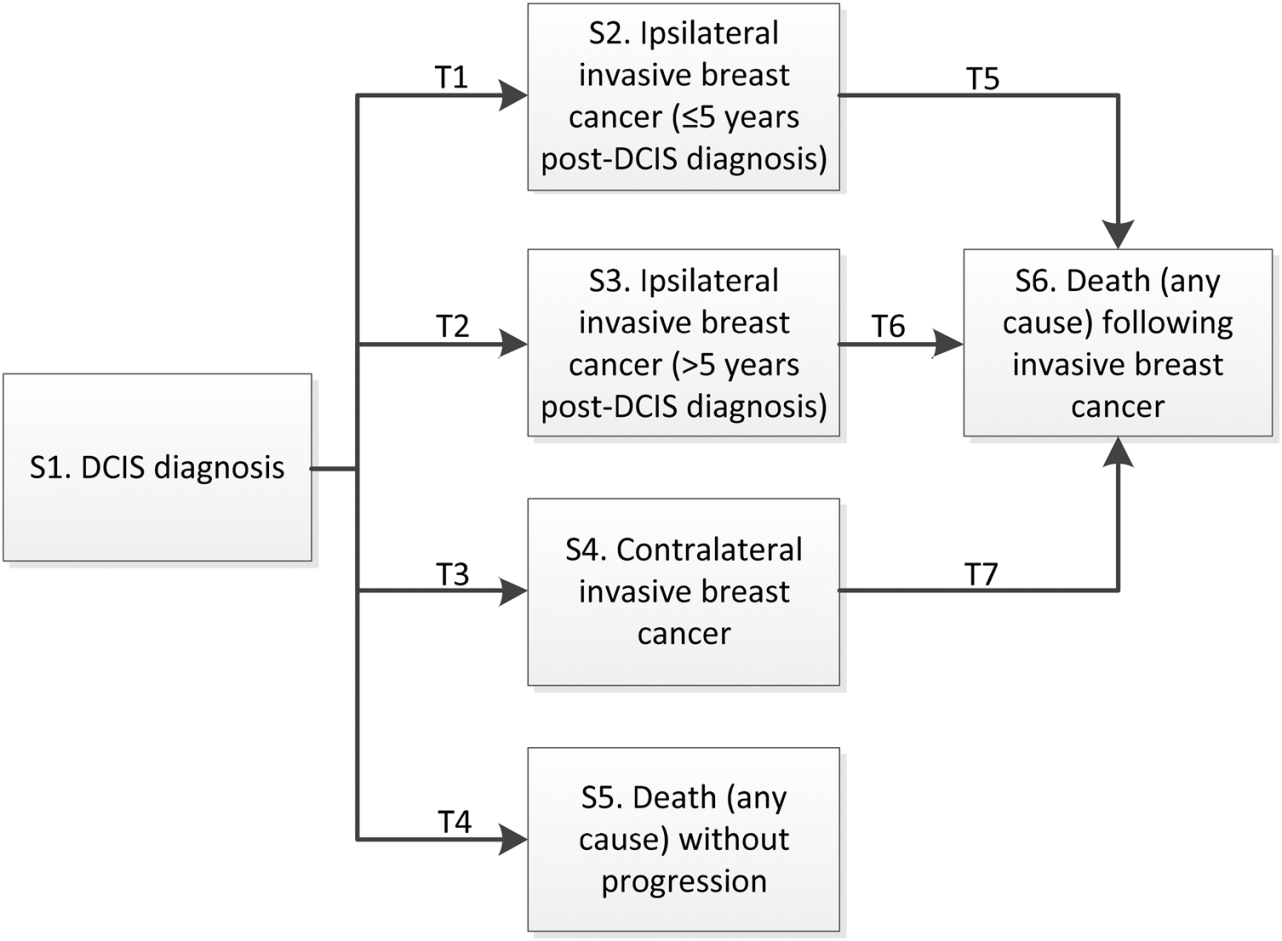
Multi-state model structure. The multi-state model structure includes six mutually exclusive health states (S1–S6) each represented by a box, and the seven transitions between each state (T1–T7). Arrows represent all possible transitions between states which were modelled. Transitions between states were modelled using multivariate Cox proportional hazards models, which assessed the impact of patient and treatment characteristics on the hazard (risk) of each event (i.e. transition from one health state to another)

The multi-state model structure includes six mutually exclusive states, and the seven transitions between each state (Fig. 2). The effects of baseline patient, disease, and treatment characteristics on each transition was assessed using multivariate Cox proportional hazard regression models. The selected covariates included age at diagnosis (40–49, 50–69, 70–74, 75–79, ≥ 80 years), diagnosis year (1992–2016), race (Hispanic and non-Hispanic white, Hispanic and non-Hispanic black, other [Asian, Native American, Pacific Islander]), grade (I, II, III), lesion size (< 2 cm, ≥ 2 cm), estrogen receptor (ER) status, and local treatment strategy (no local treatment, breast conserving surgery [BCS] only, BCS followed by radiotherapy [RT], mastectomy). Complete cases were available for all variables (age, diagnosis year, treatment strategy), except for ER status, lesion size, and race. Missing observations were imputed with the substantive model compatible fully conditional specification method using co-variables with complete cases (age, diagnosis year, treatment strategy) and outcome (time, event). This method allows greater flexibility for non-linear models such as the Cox model, in that partially observed covariates are imputed based on non-linear covariate effectsx [12]. The R package smcfcs version 1.4.0 was used.

To address possible confounding by indication, i.e. the systematic differences between patients undergoing different treatment strategies, propensity scores (PS) were calculated for each individual. The propensity score is an individual’s probability of receiving treatment given their pre-treatment characteristics (i.e. age, diagnosis year, grade, race, lesion size, ER status). As there are four treatment strategies being compared, generalized boosted regression models were used to compute PS weights which balance the distribution of selected characteristics between treatment and comparison groups. The pre-treatment characteristics listed above were used to calculate PS. The mean standardized effect size and Kolmogorov-Smirnof statistic were used to choose the optimal number of iterations to establish balance. Average treatment effect (ATE) analysis was conducted to determine the relative effectiveness of no intervention, BCS, BCS+RT, and mastectomy on average in the population. For each transition-specific Cox proportional hazards model, individuals were weighted by the inverse probability of receiving the treatment they received. Doubly robust estimation controlled for any covariates with lingering imbalances. PS analysis was conducted using the R package Twang version 1.5.

To address the violation of the proportionality assumption for some predictors in the Cox model for the transition from DCIS diagnosis to iIBC and to address the Markov assumption, time to iIBC was split at 5 years post-DCIS. Therefore the following multi-state transitions were modeled: T1. DCIS diagnosis → iIBC ≤ 5 years following diagnosis; T2. DCIS diagnosis → iIBC > 5 years following DCIS diagnosis; T3. DCIS diagnosis → cIBC; T4. DCIS diagnosis → death; T5. iIBC ≤ 5 years following diagnosis → death; T6. iIBC > 5 years following diagnosis → death; T7. cIBC → death. Intermediate lesions such as a subsequent diagnosis of DCIS during follow-up after initial DCIS are not considered in the model.

Conditional transition probabilities were computed for each treatment strategy cohort (except mastectomy) and the sub-cohort of patients with low-risk features (Hispanic and non-Hispanic white women aged 50–69 at diagnosis, with ER+, grade 1 + 2, ≤ 2 cm DCIS lesions) by building Cox models stratified by transition to compute cumulative transition hazards transformed into conditional transition probabilities using the Aalen-Johansen estimator. State occupation probabilities at different time points following DCIS diagnosis could be derived from these values. Data preparation and multi-state modeling was done using the R package mstate version 0.2.11.

PS-matched groups were also created for comparison when calculating the transition probabilities derived from the multi-state models. 1:2 matching of the n=338 individuals in the low-risk non-intervention group to each of the low-risk treatment groups was carried out using the “nearest neighbour” method in the MatchIt R package version 3.0.2. Exact matching was specified by year of diagnosis, age at diagnosis, and grade. Differences in iIBC at 5 years between low-risk PS-matched treatment groups were also evaluated using hazard ratios with 95% CIs derived from Cox proportional hazards models.

All statistical analyses were performed with R version 3.6.1 (R Foundation for Statistical Computing, Vienna, Austria).

## Results

### Patient characteristics

Table 1 shows the patient and clinicopathologic characteristics of the *N* = 85,982 individuals included in the analysis set, including *N* = 1650 who did not receive local intervention, and *N* = 17,714 patients with low-risk features (Hispanic and non-Hispanic white women aged 50–69 at diagnosis, with ER+, grade 1 + 2, ≤ 2 cm DCIS lesions). Women undergoing more invasive procedures (BCS+RT, mastectomy) were generally younger with higher-risk features (high grade, large lesion sizes).

**Table 1:** Patient and clinical-pathological characteristics

	Characteristic	No local intervention (*n* = 1,650) *n* (%)	BCS only (n = 22,698) *n* (%)	BCS+ RT (*n* = 40,265) *n* (%)	Mastectomy (*n* = 21,369) *n* (%)	Total population (*n* = 85,982) *n* (%)
Median follow-up, months (IQR)		73 (34–133)	93 (44–152)	87 (43–140)	90 (45–150)	89 (44–145)
Year of diagnosis	1992–1999	118 (7.2 %)	2739 (12.1 %)	3110 (7.7 %)	2466 (11.5 %)	8433 (9.8 %)
	2000–2010	906 (54.9 %)	12,699 (55.9 %)	22,024 (54.7 %)	11,495 (53.8 %)	47,124 (54.8 %)
	2011–2016	626 (37.9 %)	7260 (32.0 %)	15,131 (37.6 %)	7408 (34.7 %)	30,425 (35.4 %)
Age at diagnosis	40–49	323 (19.6 %)	3992 (17.6 %)	8588 (21.3 %)	6159 (28.9 %)	19,062 (22.2 %)
	50–69	826 (50.1 %)	11,375 (50.1 %)	24,386 (60.6 %)	11,268 (52.7 %)	47,855 (55.7 %)
	70–74	154 (9.3 %)	2622 (11.6 %)	3837 (9.5 %)	1677 (7.8 %)	8290 (9.6 %)
	75–79	121 (7.3 %)	2251 (9.9 %)	2324 (5.8 %)	1299 (6.1 %)	5995 (7.0 %)
	≥ 80	226 (13.7 %)	2458 (10.8 %)	1130 (2.8 %)	966 (4.5 %)	4780 (5.6 %)
Race	White	1169 (70.8 %)	18,037 (79.5 %)	31,284 (77.7 %)	16,503 (77.2 %)	66,993 (77.9 %)
	Black	239 (14.5 %)	2241 (9.9 %)	4417 (10.7 %)	2329 (10.9 %)	9226 (10.7 %)
	Other (American Indian/AK Native, Asian/Pacific Islander)	133 (8.1 %)	2172 (9.6 %)	4375 (10.9 %)	2432 (11.4 %)	9112 (10.6 %)
	Unknown	109 (6.6 %)	248 (1.1 %)	189 (0.5 %)	105 (0.5 %)	651 (0.8 %)
DCIS grade	I	364 (22.1 %)	5912 (26.0 %)	6092 (15.1 %)	2645 (12.4 %)	15013 (17.5 %)
	II	786 (47.6 %)	11,378 (50.1 %)	17,688 (43.9 %)	8470 (39.6 %)	38,322 (44.6 %)
	III	500 (30.3 %)	5408 (23.8 %)	16,485 (40.9 %)	10,254 (48.0 %)	32,647 (38.0 %)
Lesion size	< 2 cm	490 (29.7 %)	14,470 (63.6 %)	25,731 (63.9 %)	9263 (43.3 %)	49,954 (58.0%)
	2–5 cm	110 (6.7 %)	2167 (9.5 %)	5529 (13.7 %)	5099 (23.9 %)	12,905 (15.0%)
	> 5 cm	34 (2.1 %)	287 (1.3 %)	498 (1.2 %)	1560 (7.3 %)	2379 (2.8%)
	Unknown	1016 (61.6 %)	5774 (25.4 %)	8507 (21.1 %)	5447 (25.5 %)	20,744 (24.1%)
ER status	Positive/Borderline	918 (45.7 %)	11,729 (51.7 %)	25,117 (62.4 %)	11,153 (52.2 %)	48,704 (56.6 %)
	Negative	127 (6.3 %)	1232 (5.4 %)	4317 (10.7 %)	2955 (13.8 %)	8595 (10.0 %)
	Unknown	963 (48.0 %)	9737 (42.9 %)	10,831 (26.9 %)	7261 (34.0 %)	28683 (33.4 %)

*AK* Alaska,* DCIS* ductal carcinoma in-situ,* BCS* breast conserving surgery,* IQR* inter-quartile range,* ER* estrogen receptor,* REF* reference category,* RT* radiotherapy

### Annual iIBC incidence rate (1996–2016)

**Fig. 3:**
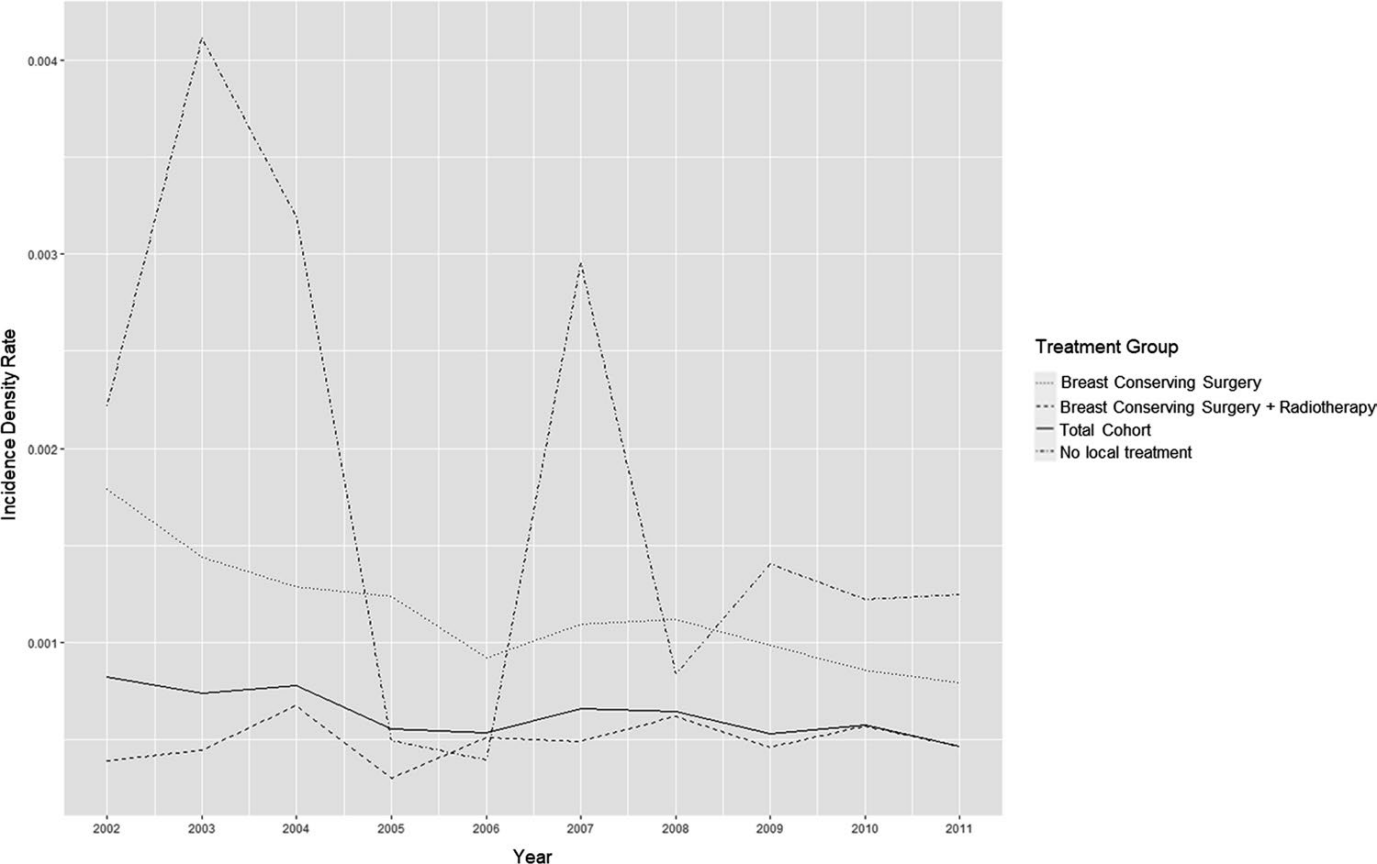
Ipsilateral invasive breast cancer (iIBC) incidence density rate (2002-2011)

Figure 3 shows the annual iIBC incidence density rate across the 2002–2011 observation period according to the person-years at risk within our cohort during each year. With the exception of the group without local treatment, there is no obvious jump in iIBC rates post-2007. This pattern remained steady across treatment cohorts.

### Transition-specific PS-weighted multivariate Cox proportional hazards models

Select baseline risk factors were shown to be highly predictive of iIBC events within the first 5 year period, with diminishing hazard for later occurring events (Table 2). Multivariate-adjusted PS-weighted models showed that women aged 40–49 at diagnosis had a statistically significantly higher risk of subsequent iIBC within 5 years compared to women aged 50–69 (Hazard ratio (HR) 1.86, 95% Confidence Interval (CI) 1.34–2.57). Grade 3 lesions also carried a higher risk compared to grade 2 (HR 1.42, 95% CI 1.05–1.91). This significant effect of high grade was not observed for events occurring after 5 years (HR 1.00, 95% CI 0.73–1.38). Lesion size ≥ 2 cm (HR 1.66, 95% CI 1.23–2.25), and black race (HR 2.52, 95% CI 1.83–3.48 compared to white race) were also predictive of subsequent iIBC events within 5 years and after 5 years (Table 2). ER+ status did not have a statistically significant association with iIBC risk for any time period. Age groups ≥ 70 years did not show a statistically significant different HR of iIBC ≤ 5 years compared to age 50–69; nor did grade 1 compared to grade 2 (Table 2).

**Table 2:** Propensity score-weighted Cox proportional hazards models

		T1 (diagnosis → iIBC ≤ 5) HR (95% CI)	T2 (diagnosis → iIBC > 5) HR (95% CI)	T3 (diagnosis → cIBC) HR (95% CI)	T4 (diagnosis → death) HR (95% CI)	T5 (iIBC ≤ 5 → death) HR (95% CI)	T6 (iIBC > 5 → death) HR (95% CI)	T7 (cIBC → death) HR (95% CI)
Local treatment	BCS+RT	REF	REF	REF	REF	REF	REF	REF
	Mastectomy	0.50 (0.39–0.65)	0.21 (0.16–0.26)	1.18 (1.07–1.31)	1.14 (1.08–1.20)	1.30 (0.74–2.30)	1.31 (0.68–2.52)	0.96 (0.75–1.23)
	BCS	3.14 (2.70–3.67)	1.35 (1.20–1.53)	1.05 (0.95–1.16)	1.19 (1.13–1.25)	1.05 (0.70–1.58)	0.99 (0.70–1.39)	1.01 (0.78–1.23)
	No local treatment	4.26 (3.12–5.81)	1.69 (1.19–2.41)	1.17 (0.87–1.57)	1.55 (1.34–1.78)	3.32 (1.31–8.45)	1.01 (0.38–2.70)	1.81 (1.06–3.09)
Year of diagnosis		1.02 (0.98–1.05)	0.97 (0.94–1.01)	1.00 (0.98–1.01)	1.00 (0.99–1.01)	1.03 (0.97–1.09)	0.98 (0.86 –1.12)	1.02 (0.97–1.07)
Age at DCIS diagnosis	50–69	REF	REF	REF	REF	REF	REF	REF
	40–49	1.86 (1.34–2.57)	1.37 (1.02–1.83)	0.76 (0.63–0.92)	0.31 (0.24–0.39)	1.29 (0.55–3.07)	1.20 (0.50–2.85)	1.23 (0.68–2.23)
	70–74	1.01 (0.59–1.74)	1.20 (0.74–1.95)	1.40 (1.09–1.80)	3.23 (2.84–3.68)	4.97 (2.08–11.88)	2.44 (0.98–6.05)	2.08 (1.19–3.64)
	75–79	0.95 (0.61–1.46)	0.97 (0.75–1.25)	1.02 (0.76–1.37)	5.96 (5.28–6.72)	4.46 (2.11–9.44)	4.25 (2.28–7.90)	2.96 (1.54–5.68)
	≥ 80	1.14 (0.80–1.63)	0.62 (0.44–0.87)	1.06 (0.74–1.51)	10.84 (9.78–12.02)	6.24 (3.26–11.93)	6.76 (3.12–14.64)	5.86 (3.73–9.22)
Grade	2	REF	REF	REF	REF	REF	REF	REF
	1	0.87 (0.58–1.32)	1.04 (0.74–1.44)	0.84 (0.69–1.02)	1.13 (1.02–1.24)	0.72 (0.29–1.78)	0.94 (0.41–2.14)	1.26 (0.82–1.93)
	3	1.42 (1.05–1.91)	1.00 (0.73–1.38)	0.82 (0.68–1.02)	1.10 (0.99–1.22)	1.23 (0.66–2.32)	1.29 (0.69–2.42)	0.70 (0.45–1.09)
Race	Caucasian	REF	REF	REF	REF	REF	REF	REF
	African American	2.52 (1.83–3.48)	1.79 (1.25–2.55)	1.12 (0.89–1.42)	1.37 (1.21–1.54)	1.47 (0.71–3.07)	1.51 (0.68–3.39)	0.97 (0.58–1.62)
	Other	1.46 (0.84–2.55)	1.54 (1.00–2.36)	1.07 (0.82–1.39)	0.75 (0.64–0.88)	0.46 (0.15–1.40)	1.77 (0.64–4.95)	0.66 (0.26–1.69)
Lesion size	< 2 cm	REF	REF	REF	REF	REF	REF	REF
	≥ 2 cm	1.66 (1.23–2.25)	1.38 (1.00–2.36)	1.08 (0.90–1.30)	1.18 (1.06–1.30)	1.75 (1.02–3.01)	1.80 (0.82–3.93)	1.28 (0.85–1.91)
ER status	Negative	REF	REF	REF	REF	REF	REF	REF
	Positive	0.76 (0.53–1.10)	1.51 (0.98–2.33)	1.13 (0.87–1.46)	0.94 (0.83–1.06)	0.40 (0.19–0.85)	0.51 (0.29–0.90)	0.67 (0.36–1.25)

*CI* confidence interval,* cIBC* contralateral invasive breast cancer,* DCIS* ductal carcinoma in-situ,* BCS* breast conserving surgery,* HR* hazard ratio,* iIBC* ipsilateral invasive breast cancer,* IQR* inter-quartile range,* ER* estrogen receptor,* REF* reference category,* RT* radiotherapy

Baseline characteristics of the primary DCIS did not demonstrate any statistically significant relationship with cIBC events, with the exception of age 70–74 which carried a higher hazard of cIBC events compared to age 50–69 (HR 1.26, 95% CI 1.11–1.42) (Table 2).

### Multi-state modeling

**Fig. 4:**
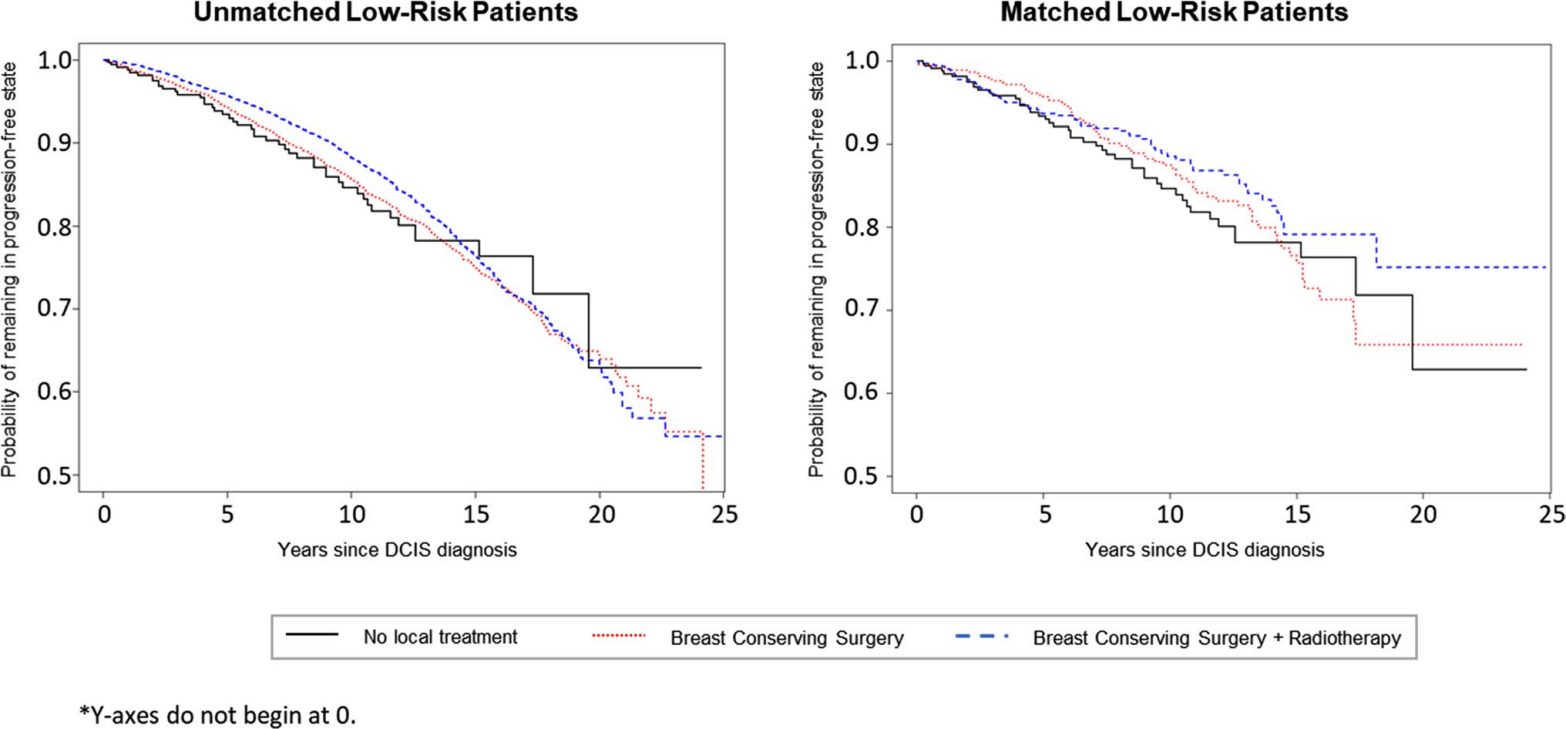
Progression-free state occupancy probabilities for patients with low-risk features

State occupancy probabilities for the “progression-free” state calculated from the multi-state models are visualized in Fig. 4 for the different treatment modalities for patients in the low-risk subgroup. All other transition probabilities calculated from the multi-state models are visualized in Supplementary Fig. 1; the distance between two curves represents the probability of being in a specific state at a specific time point (state occupation probability). Time-dependent transition probabilities and accompanying standard errors are listed in Supplementary Table 1–8. For low-risk women not receiving local treatment, the probability of being alive and remaining iIBC-free at 5 years was 95.5% (95% CI 87.5–98.4%) and 89.2% (95% CI 78.2–94.7%) at 10 years. The probability of experiencing an iIBC as first event at 5 years was 0.92% (95% CI 0.00–1.95%) and 3.02% (95% CI 0.00–6.05%) at 10 years. In the same cohort of low-risk women, matched according to PS and patient characteristics, the probability of experiencing an iIBC at 5 years was 0.88% (95% CI 0.10–1.66%) following BCS, and 0.35% (95% CI 0.00–0.80%) following BCS+RT. The 10 year probability was 2.48% (95% CI 0.82–4.11%) and 0.58% (95% CI 0.00–1.39%) respectively for BCS and BCS+RT. All transition probabilities in PS-matched groups are listed in Supplementary Tables 1–8. No statistically significant differences in iIBC at 5 years between low-risk PS-matched treatment groups were detected (BCS vs. AS: HR 0.83, 95% CI 0.19–3.48; BCS+RT vs. AS: HR 0.75, 95% CI 0.13–4.49).

## Discussion

This analysis applied real-world cancer registry data from n=85,982 women diagnosed with primary DCIS. The excellent iIBC-free survival observed at 5 and 10 years for the women in this cohort with low-risk features is an important confirmation that an active surveillance strategy could be safe and feasible compared to standard interventional treatment. For those with low-risk features (Hispanic and non-Hispanic white women aged 50–69 at diagnosis, with ER+, grade 1 + 2, ≤ 2 cm DCIS lesions) who did not receive local treatment, their prognosis remained comparable to their matched counterparts who received surgery with or without radiotherapy. The observed 10-year probability of iIBC at 3.0%, as well as the combined risk of contralateral and ipsilateral IBC remains well within the 10-year population-wide age-specific probability of developing IBC for US women (range 2.3–3.9%). [13]

Improving the understanding of the disease process after diagnosis and treatment of primary DCIS remains an important undertaking. Through the development of multi-state models using real-world data, we were able to provide insight into how patients transition from DCIS diagnosis to iIBC or cIBC across treatment strategies. Multi-state modeling provides an advantageous approach over typical time-to-event modeling techniques as it allowed us to visualize competing event risks, and to understand what happens after an intermediate event such as an IBC. Across treatment strategies there were similar probabilities of dying without an IBC, with comparatively very low probabilities of death following IBC (Supplementary Fig. 1).

Previous studies have attempted to simulate various possibilities of the natural history of DCIS, without demarcating subgroups based on risk of subsequent breast events [14]. This is the first study to explicitly model the disease process for women with features deemed to make them low-risk for subsequent iIBC, for whom an active surveillance strategy is targeted towards. We provide evidence beyond previously published studies which provided limited direct comparison of no locoregional treatment and standard surgical strategies. Ryser et al. recently conducted a study on cancer outcomes in DCIS patients without locoregional treatment identified in the SEER dataset. When analyzing their low-risk subgroup (non-high grade, ER/PR+, > 40 years at diagnosis) in a competing risk analysis, the 7.5-year cumulative incidence of iIBC was 5.9% (95% CI 2.3–9.5%) [15]. In our analysis, the subgroup of low-risk women is further limited to women aged 50–69 at diagnosis, with small (< 2 cm) lesions. We additionally limit this selection to Hispanic and non-Hispanic White women, as our multi-state model revealed Black race to be a strong marker of iIBC ≤ 5 years post-DCIS. As cancer health disparities in racial and ethnic groups in the United States are well-established, in this analysis we do not designate race as a biological risk factor [16]. Further analysis into the systemic disadvantage and structural inequalities in screening and follow-up care which contribute to poorer health outcomes for women in minority racial and ethnic groups diagnosed with DCIS is warranted.

The SEER dataset is rich in clinico-pathological information and socio-demographic information which helps us to understand who is more likely to receive certain treatment modalities and how this impacts their health outcomes. However, despite SEER being one of the widely used cancer registries for observational research, its use is not without its possible pitfalls. The potential impact of misclassification of surgery and radiation for women who did not receive treatment should be confirmed by careful review of medical records or by patient interview. While SEER records the most invasive surgical procedure on the primary site, it is possible that some women diagnosed with DCIS at one institution sought surgical and/or radiation treatment at another institution not within the same SEER registry catchment area. Nevertheless, analyses comparing agreement between SEER data and Medicare claims for receipt of RT demonstrated that SEER reliably identified individuals who received treatment for in situ female breast events [17]. Beyond potential misclassification of treatment, the Ryser study was critiqued as having artificially low estimates of iIBC incidence, especially for cases diagnosed before changes to SEER coding of “recurrences” in 2007 [15, 18]. The SEER program collects data on subsequent primary cancers, but does not record information on cancer recurrences. Indeed, a diagnosis of a subsequent invasive breast cancer following DCIS can be described either as a loco-regional invasive recurrence or a new primary cancer, and language to describe this phenomena has not been consistent. In order to understand the impact of changes in SEER coding rules in 2007 which may have led to the earlier under-reporting of subsequent iIBC following DCIS, we calculated the annual iIBC incidence density rate across the 2002–2011 observation period according to the person-time at risk within our cohort during each year. The group without local treatment showed significant variation over time, while the pattern remained steady for the cohort as a whole. This is an important observation to understand relative treatment effects (Fig. 3).

Previous studies of IBC have made attempts to distinguish new primary tumors from true recurrences after IBC, with consistent reporting that true recurrences occur sooner than new primary tumors [19-21]. We identified time dependencies for many covariates in our Cox models. This led us to splitting iIBC into two states, distinguished by events that occurred within, or following, 5 years after DCIS diagnosis. We observed a strong association between high grade and earlier ipsilateral invasive events (occurring within 5 years). The same association was not observed for events occurring after 5 years. It is possible that this is a reflection of the clonal relationship of the primary DCIS and any subsequent iIBCs; we can hypothesize that iIBC events occurring more than 5 years after the primary DCIS are likely to be unrelated, new primary tumors. Previously published information on IBC after DCIS combined with our evidence on the time-dependency of DCIS grade can inform decisions on appropriate follow-up length for future studies concerning treatment approaches for primary DCIS.

To explore the relative treatment effects on iIBC within 5 years of DCIS diagnosis for women with low-risk features, we looked at treatment-specific hazard ratios. Women with no local treatment were matched 1:2 with women treated with surgery (BCS ± RT) according to PS, and by year of diagnosis, age at diagnosis, and grade (all women considered low-risk had ER+ lesions < 2 cm and were (non)-Hispanic white). Hazard ratios showed a protective effect for surgical interventions (HR < 1) but this was non-significant in all cases.

It is well-known that the diagnosis of DCIS is associated with an increased risk of breast cancer. Retrospective observational registry studies continue to confirm this in different screen-detected DCIS populations [22]. However, for women with low-risk features, this risk is likely to be well-managed with an active surveillance strategy where bi-annual physical examinations and annual mammography allow the lesion to be closely monitored. If a woman receives local treatment for DCIS, the likelihood of a subsequent iIBC remains low. However, any subsequent loco-regional iIBC events in a previously irradiated breast will be more difficult to treat locally with re-irradiation due to increased risk of skin and subcutaneous toxicity because re-irradiation will exceed the maximum tolerable dose of radiotherapy of the skin and subcutaneous tissue. Irreversible radiation-induced fibrosis and radionecrosis hinders the efficacy of systemic chemotherapy [23]. Treatment-related complications are further compounded by the emotional and economic toll that initial local treatment represents [5]. In a recent study on treatment preferences for screen-detected DCIS, patients valued active monitoring over standard interventional treatment [24]. This was largely influenced by the risk of progression: a 10% risk of progression at 10 years was deemed an acceptable trade-off to avoid possible side-effects from surgery or radiotherapy. Compared to the observed iIBC risk at 10 years in women with low-risk characteristics who did not receive local treatment at 3%, this provides further evidence of patients’ willingness to be followed under a demonstrably safe active surveillance strategy.

## Conclusion

As physicians treating women with low-risk DCIS await results from prospective trials on active surveillance, there is value in harnessing real-world evidence from cancer registries to support present-day decision-making for possible non-intervention in (low-risk) DCIS. With multi-state models, it is possible to visualize, quantify, and compare competing breast event risks for different treatment and risk groups. Evaluating time dependencies of prognostic factors in the models also allowed for the understanding of the relationship between subsequent iIBCs and the primary DCIS. Replacing conventional invasive treatment with active surveillance in this good prognosis population could improve women’s well-being during the remaining (progression-free) survival time without resulting in significantly poorer disease outcome. This is an important factor to consider when making an informed treatment decision in this patient population. Capturing the full impact of possible treatment strategies over a patient’s lifetime involves integrating health outcomes, health-related quality of life, patient and provider preference, as well as direct and indirect costs. In this study we provide the first set of information to help model progression outcomes and transitions between health states. This model can easily be extended to integrate cost and quality of life data points, so that researchers can model the potential cost-utility of new disease management strategies for this specific cohort of low-risk DCIS patients.

## Supplementary Information

Below is the link to the electronic supplementary material.

(pdf 172 kb)

## Data Availability

The data that support the findings of this study are openly available online in the Surveillance, Epidemiology, and End Results Program (SEER) at https://seer.cancer.gov/.
