# Supplementary material for: Treating (low-risk) DCIS patients: What can we learn from real-world cancer registry evidence?
Source: Breast Cancer Res Treat. 2021 Jan 3;187(1):187–96. doi: 10.1007/s10549-020-06042-1 (PMC8062323; doi:10.1007/s10549-020-06042-1)
Supplement: Supplementary file 1 — (pdf 172 kb) [file 10549_2020_6042_MOESM1_ESM.pdf]

Online Only Supplements Accompanying:
Treating (low-risk) DCIS Patients: What can we learn from real-world cancer registry evidence?

Danalyn Byng,[1,2] Valesca P. Retèl,[1,2] Michael Schaapveld,[1] Jelle Wesseling,[3] Wim H. van Harten[1,2]

[1]Division of Psychosocial Research and Epidemiology, The Netherlands Cancer Institute-Antoni van Leeuwenhoek Hospital, Plesmanlaan 121, 1066 CX Amsterdam, The Netherlands.
[2]Health Technology and Services Research Department, Technical Medical Centre, University of Twente, P.O. Box 217, 7500 AE Enschede, The Netherlands.
[3]Division of Molecular Pathology, The Netherlands Cancer Institute-Antoni van Leeuwenhoek Hospital, Plesmanlaan 121, 1066 CX Amsterdam, The Netherlands.

Contents

Supplementary Table 1. Probability of Remaining in DCIS State (state occupancy probability of "progression-free survival") ... 1

Supplementary Table 2. Transition 1: probability of transitioning from DCIS state to ipsilateral invasive breast cancer ≤5 years post-diagnosis ... 2

Supplementary Table 3. Transition 2: probability of transitioning from DCIS state to ipsilateral invasive breast cancer >5 years post-diagnosis ... 3

Supplementary Table 4. Transition 3: probability of transitioning from DCIS state to contralateral invasive breast cancer ... 4

Supplementary Table 5. Transition 4: probability of transitioning from DCIS state to death without progression ... 5

Supplementary Table 6. Transition 5: probability of transitioning from ipsilateral invasive breast cancer (≤5 years post-DCIS diagnosis) to death ... 6

Supplementary Table 7. Transition 6: probability of transitioning from ipsilateral invasive breast cancer (>5 years post-DCIS diagnosis) to death ... 7

Supplementary Table 8. Transition 7: probability of transitioning from contralateral invasive breast cancer to death ... 8

Supplementary Figure 1: State occupation probabilities and transition probabilities for women with low-risk features ... 9

Supplementary Table 1. Probability of Remaining in DCIS State (state occupancy probability of “progression-free survival”)

	**No treatment**		**All low-risk DCIS patients**						**Propensity Score-Matched 1:2 low-risk DCIS patients**					
			**BCS**		**BCS+RT**		**MAST**		**BCS**		**BCS+RT**		**MAST**	
	Pr.	SE	Pr.	SE	Pr.	Pr.	Pr.	SE	Pr.	SE	Pr.	Pr.	Pr.	SE
Year 0	1.000	0.000	1.000	0.000	1.000	1.000	1.000	0.000	1.000	0.000	1.000	1.000	1.000	0.000
Year 1	0.988	0.006	0.990	0.001	0.995	0.001	0.986	0.002	0.990	0.004	0.993	0.003	0.985	0.006
Year 2	0.975	0.009	0.980	0.002	0.988	0.001	0.979	0.003	0.987	0.005	0.974	0.007	0.982	0.006
Year 3	0.958	0.011	0.969	0.002	0.978	0.002	0.971	0.003	0.976	0.006	0.960	0.008	0.977	0.007
Year 4	0.954	0.012	0.960	0.003	0.967	0.002	0.962	0.004	0.970	0.007	0.948	0.009	0.972	0.008
Year 5	0.934	0.015	0.942	0.003	0.957	0.002	0.951	0.005	0.957	0.009	0.934	0.011	0.955	0.011
Year 6	0.916	0.017	0.926	0.004	0.945	0.003	0.938	0.005	0.943	0.010	0.932	0.011	0.948	0.011
Year 7	0.902	0.018	0.908	0.004	0.932	0.003	0.925	0.006	0.918	0.013	0.919	0.012	0.941	0.012
Year 8	0.882	0.021	0.892	0.005	0.918	0.004	0.914	0.006	0.898	0.015	0.916	0.013	0.937	0.013
Year 9	0.859	0.023	0.873	0.005	0.902	0.004	0.903	0.007	0.885	0.016	0.896	0.015	0.932	0.014
Year 10	0.846	0.024	0.855	0.006	0.882	0.004	0.887	0.008	0.871	0.017	0.881	0.016	0.911	0.017

Supplementary Table 2. Transition 1: probability of transitioning from DCIS state to ipsilateral invasive breast cancer ≤5 years post-diagnosis

	**No treatment**		**All low-risk DCIS patients**						**Propensity Score-Matched 1:2 low-risk DCIS patients**					
			**BCS**		**BCS+RT**		**MAST**		**BCS**		**BCS+RT**		**MAST**	
	Pr.	SE	Pr.	SE	Pr.	SE	Pr.	SE	Pr.	SE	Pr.	SE	Pr.	SE
Year 0	0.000	0.000	0.000	0.000	0.000	0.000	0.000	0.000	0.000	0.000	0.000	0.000	0.000	0.000
Year 1	0.009	0.005	0.004	0.001	0.000	0.000	0.001	0.001	0.003	0.002	0.000	0.000	0.000	0.000
Year 2	0.009	0.005	0.008	0.001	0.000	0.000	0.001	0.001	0.003	0.002	0.003	0.002	0.000	0.000
Year 3	0.009	0.005	0.011	0.001	0.002	0.000	0.001	0.001	0.007	0.003	0.003	0.002	0.002	0.002
Year 4	0.009	0.005	0.013	0.002	0.003	0.001	0.002	0.001	0.007	0.003	0.003	0.002	0.002	0.002
Year 5	0.009	0.005	0.017	0.002	0.004	0.001	0.003	0.001	0.009	0.004	0.003	0.002	0.005	0.003

BCS: breast conserving surgery; BCS+RT: breast conserving surgery + radiotherapy; MAST: mastectomy; Pr.: probability; SE: standard error

Supplementary Table 3. Transition 2: probability of transitioning from DCIS state to ipsilateral invasive breast cancer >5 years post-diagnosis

	**No treatment**		**All low-risk DCIS patients**						**Propensity Score-Matched 1:2 low-risk DCIS patients**					
			**BCS**		**BCS+RT**		**MAST**		**BCS**		**BCS+RT**		**MAST**	
	Pr.	SE	Pr.	SE	Pr.	SE	Pr.	SE	Pr.	SE	Pr.	SE	Pr.	SE
Year 0	0.000	0.000	0.000	0.000	0.000	0.000	0.000	0.000	0.000	0.000	0.000	0.000	0.000	0.000
Year 1	0.000	0.000	0.000	0.000	0.000	0.000	0.000	0.000	0.000	0.000	0.000	0.000	0.000	0.000
Year 2	0.000	0.000	0.000	0.000	0.000	0.000	0.000	0.000	0.000	0.000	0.000	0.000	0.000	0.000
Year 3	0.000	0.000	0.000	0.000	0.000	0.000	0.000	0.000	0.000	0.000	0.000	0.000	0.000	0.000
Year 4	0.000	0.000	0.000	0.000	0.000	0.000	0.000	0.000	0.000	0.000	0.000	0.000	0.000	0.000
Year 5	0.000	0.000	0.000	0.000	0.000	0.000	0.000	0.000	0.000	0.000	0.000	0.000	0.000	0.000
Year 6	0.004	0.004	0.005	0.001	0.003	0.001	0.001	0.001	0.004	0.003	0.000	0.000	0.000	0.000
Year 7	0.004	0.004	0.010	0.002	0.005	0.001	0.001	0.001	0.015	0.006	0.003	0.003	0.000	0.000
Year 8	0.015	0.008	0.014	0.002	0.007	0.001	0.001	0.001	0.017	0.006	0.006	0.004	0.000	0.000
Year 9	0.015	0.008	0.020	0.002	0.009	0.001	0.001	0.001	0.020	0.007	0.006	0.004	0.000	0.000
Year 10	0.021	0.010	0.021	0.002	0.013	0.002	0.002	0.001	0.025	0.008	0.006	0.004	0.005	0.005

BCS: breast conserving surgery; BCS+RT: breast conserving surgery + radiotherapy; MAST: mastectomy; Pr.: probability; SE: standard error

Supplementary Table 4. Transition 3: probability of transitioning from DCIS state to contralateral invasive breast cancer

	**No treatment**		**All low-risk DCIS patients**						**Propensity Score-Matched 1:2 low-risk DCIS patients**					
			**BCS**		**BCS+RT**		**MAST**		**BCS**		**BCS+RT**		**MAST**	
	Pr.	SE	Pr.	SE	Pr.	SE	Pr.	SE	Pr.	SE	Pr.	SE	Pr.	SE
Year 0	0.000	0.000	0.000	0.000	0.000	0.000	0.000	0.000	0.000	0.000	0.000	0.000	0.000	0.000
Year 1	0.003	0.003	0.004	0.001	0.004	0.001	0.011	0.002	0.007	0.003	0.007	0.003	0.011	0.005
Year 2	0.009	0.005	0.006	0.001	0.006	0.001	0.013	0.002	0.007	0.003	0.010	0.004	0.011	0.005
Year 3	0.013	0.006	0.009	0.001	0.010	0.001	0.015	0.002	0.010	0.004	0.017	0.005	0.011	0.005
Year 4	0.013	0.006	0.013	0.002	0.014	0.001	0.018	0.003	0.016	0.005	0.023	0.006	0.014	0.006
Year 5	0.021	0.009	0.017	0.002	0.016	0.001	0.022	0.003	0.018	0.006	0.028	0.007	0.019	0.007
Year 6	0.025	0.010	0.021	0.002	0.020	0.002	0.024	0.003	0.026	0.007	0.028	0.007	0.023	0.008
Year 7	0.035	0.012	0.024	0.002	0.023	0.002	0.028	0.004	0.033	0.008	0.028	0.007	0.026	0.008
Year 8	0.040	0.013	0.028	0.003	0.027	0.002	0.030	0.004	0.039	0.009	0.028	0.007	0.031	0.009
Year 9	0.040	0.013	0.029	0.003	0.029	0.002	0.033	0.004	0.039	0.009	0.024	0.007	0.035	0.010
Year 10	0.046	0.014	0.032	0.003	0.034	0.002	0.037	0.004	0.039	0.009	0.024	0.007	0.035	0.010

BCS: breast conserving surgery; BCS+RT: breast conserving surgery + radiotherapy; MAST: mastectomy; Pr.: probability; SE: standard error

Supplementary Table 5. Transition 4: probability of transitioning from DCIS state to death without progression

	No treatment		All low-risk DCIS patients						Propensity Score-Matched 1:2 low-risk DCIS patients					
			BCS		BCS+RT		MAST		BCS		BCS+RT		MAST	
	Pr.	SE	Pr.	SE	Pr.	SE	Pr.	SE	Pr.	SE	Pr.	SE	Pr.	SE
Year 0	0.000	0.000	0.000	0.000	0.000	0.000	0.000	0.000	0.000	0.000	0.000	0.000	0.000	0.000
Year 1	0.000	0.000	0.002	0.001	0.000	0.000	0.002	0.001	0.000	0.000	0.000	0.000	0.005	0.003
Year 2	0.006	0.005	0.006	0.001	0.005	0.001	0.007	0.002	0.004	0.002	0.012	0.005	0.007	0.004
Year 3	0.020	0.008	0.010	0.001	0.010	0.001	0.013	0.002	0.007	0.004	0.018	0.006	0.009	0.005
Year 4	0.024	0.009	0.014	0.002	0.015	0.001	0.018	0.003	0.007	0.004	0.024	0.006	0.012	0.005
Year 5	0.032	0.010	0.023	0.002	0.022	0.002	0.024	0.003	0.016	0.006	0.032	0.008	0.021	0.008
Year 6	0.040	0.012	0.030	0.003	0.027	0.002	0.035	0.004	0.018	0.006	0.035	0.008	0.025	0.008
Year 7	0.045	0.013	0.039	0.003	0.035	0.002	0.043	0.005	0.026	0.007	0.045	0.010	0.028	0.009
Year 8	0.050	0.014	0.048	0.003	0.042	0.003	0.053	0.005	0.035	0.009	0.045	0.010	0.028	0.009
Year 9	0.068	0.017	0.057	0.004	0.053	0.003	0.058	0.006	0.041	0.010	0.065	0.012	0.028	0.009
Year 10	0.068	0.017	0.067	0.004	0.064	0.003	0.068	0.006	0.048	0.011	0.080	0.014	0.044	0.013

BCS: breast conserving surgery; BCS+RT: breast conserving surgery + radiotherapy; MAST: mastectomy; Pr.: probability; SE: standard error

Supplementary Table 6. Transition 5: probability of transitioning from ipsilateral invasive breast cancer (≤5 years post-DCIS diagnosis) to death

	No treatment		All low-risk DCIS patients						Propensity Score-Matched 1:2 low-risk DCIS patients					
			BCS		BCS+RT		MAST		BCS		BCS+RT		MAST	
	Pr.	SE	Pr.	SE	Pr.	SE	Pr.	SE	Pr.	SE	Pr.	SE	Pr.	SE
Year 0	0.000	0.000	0.000	0.000	0.000	0.000	0.000	0.000	0.00	0.00	0.000	0.000	0.000	0.000
Year 1	0.000	0.000	0.000	0.000	0.000	0.000	0.000	0.000	0.00	0.00	0.000	0.000	0.000	0.000
Year 2	0.000	0.000	0.036	0.034	0.000	0.000	0.000	0.000	0.00	0.00	0.000	0.000	0.000	0.000
Year 3	0.000	0.000	0.036	0.034	0.111	0.099	0.000	0.000	0.00	0.00	0.500	0.250	0.000	0.000
Year 4	0.000	0.000	0.052	0.037	0.111	0.099	0.000	0.000	0.00	0.00	0.500	0.250	0.000	0.000
Year 5	0.000	0.000	0.081	0.041	0.111	0.099	0.000	0.000	0.00	0.00	0.500	0.250	0.000	0.000
Year 6	0.000	0.000	0.137	0.047	0.111	0.099	0.000	0.000	0.00	0.00	0.500	0.250	0.000	0.000
Year 7	0.000	0.000	0.165	0.050	0.111	0.099	0.000	0.000	0.00	0.00	0.500	0.250	0.000	0.000
Year 8	0.000	0.000	0.194	0.052	0.111	0.099	0.000	0.000	0.20	0.16	0.500	0.250	0.000	0.000
Year 9	0.000	0.000	0.194	0.052	0.111	0.099	0.500	0.250	0.20	0.16	0.500	0.250	0.000	0.000
Year 10	0.000	0.000	0.211	0.053	0.111	0.099	0.500	0.250	0.20	0.16	0.500	0.250	0.000	0.000

BCS: breast conserving surgery; BCS+RT: breast conserving surgery + radiotherapy; MAST: mastectomy; Pr.: probability; SE: standard error

Supplementary Table 7. Transition 6: probability of transitioning from ipsilateral invasive breast cancer (>5 years post-DCIS diagnosis) to death

	**No treatment**		**All low-risk DCIS patients**						**Propensity Score-Matched 1:2 low-risk DCIS patients**					
			**BCS**		**BCS+RT**		**MAST**		**BCS**		**BCS+RT**		**MAST**	
	Pr.	SE	Pr.	SE	Pr.	SE	Pr.	SE	Pr.	SE	Pr.	SE	Pr.	SE
Year 0	0.000	0.000	0.000	0.000	0.000	0.000	0.000	0.000	0.000	0.000	0.000	0.000	0.000	0.000
Year 1	0.000	0.000	0.000	0.000	0.000	0.000	0.000	0.000	0.000	0.000	0.000	0.000	0.000	0.000
Year 2	0.000	0.000	0.000	0.000	0.000	0.000	0.000	0.000	0.000	0.000	0.000	0.000	0.000	0.000
Year 3	0.000	0.000	0.000	0.000	0.000	0.000	0.000	0.000	0.000	0.000	0.000	0.000	0.000	0.000
Year 4	0.000	0.000	0.000	0.000	0.000	0.000	0.000	0.000	0.000	0.000	0.000	0.000	0.000	0.000
Year 5	0.000	0.000	0.000	0.000	0.000	0.000	0.000	0.000	0.000	0.000	0.000	0.000	0.000	0.000
Year 6	0.000	0.000	0.000	0.000	0.000	0.000	0.000	0.000	0.000	0.000	0.000	0.000	0.000	0.000
Year 7	0.000	0.000	0.000	0.000	0.045	0.043	0.000	0.000	0.000	0.000	0.000	0.000	0.000	0.000
Year 8	0.000	0.000	0.023	0.023	0.045	0.043	0.000	0.000	0.000	0.000	0.000	0.000	0.000	0.000
Year 9	0.000	0.000	0.057	0.032	0.073	0.050	0.000	0.000	0.125	0.109	0.000	0.000	0.000	0.000
Year 10	0.000	0.000	0.088	0.038	0.099	0.055	0.000	0.000	0.250	0.142	0.000	0.000	0.000	0.000

BCS: breast conserving surgery; BCS+RT: breast conserving surgery + radiotherapy; MAST: mastectomy; Pr.: probability; SE: standard error

Supplementary Table 8. Transition 7: probability of transitioning from contralateral invasive breast cancer to death

	**No treatment**		**All low-risk DCIS patients**						**Propensity Score-Matched 1:2 low-risk DCIS patients**					
			**BCS**		**BCS+RT**		**MAST**		**BCS**		**BCS+RT**		**MAST**	
	Pr.	SE	Pr.	SE	Pr.	SE	Pr.	SE	Pr.	SE	Pr.	SE	Pr.	SE
Year 0	0.000	0.000	0.000	0.000	0.000	0.000	0.000	0.000	0.000	0.000	0.000	0.000	0.000	0.000
Year 1	0.000	0.000	0.000	0.000	0.000	0.000	0.000	0.000	0.000	0.000	0.000	0.000	0.000	0.000
Year 2	0.000	0.000	0.040	0.038	0.000	0.000	0.000	0.000	0.000	0.000	0.000	0.000	0.000	0.000
Year 3	0.000	0.000	0.040	0.038	0.017	0.017	0.000	0.000	0.000	0.000	0.000	0.000	0.000	0.000
Year 4	0.000	0.000	0.040	0.038	0.029	0.021	0.000	0.000	0.000	0.000	0.000	0.000	0.000	0.000
Year 5	0.250	0.188	0.040	0.038	0.058	0.026	0.025	0.024	0.000	0.000	0.000	0.000	0.000	0.000
Year 6	0.250	0.188	0.040	0.038	0.075	0.028	0.025	0.024	0.000	0.000	0.000	0.000	0.000	0.000
Year 7	0.250	0.188	0.086	0.043	0.075	0.028	0.025	0.024	0.000	0.000	0.000	0.000	0.000	0.000
Year 8	0.357	0.185	0.096	0.044	0.098	0.030	0.025	0.024	0.077	0.071	0.000	0.000	0.000	0.000
Year 9	0.357	0.185	0.145	0.046	0.113	0.031	0.066	0.037	0.077	0.071	0.125	0.109	0.000	0.000
Year 10	0.357	0.185	0.203	0.049	0.120	0.032	0.107	0.045	0.077	0.071	0.125	0.109	0.000	0.000

BCS: breast conserving surgery; BCS+RT: breast conserving surgery + radiotherapy; MAST: mastectomy; Pr.: probability; SE: standard error

Supplementary Figure 1: State occupation probabilities and transition probabilities for women with low-risk features

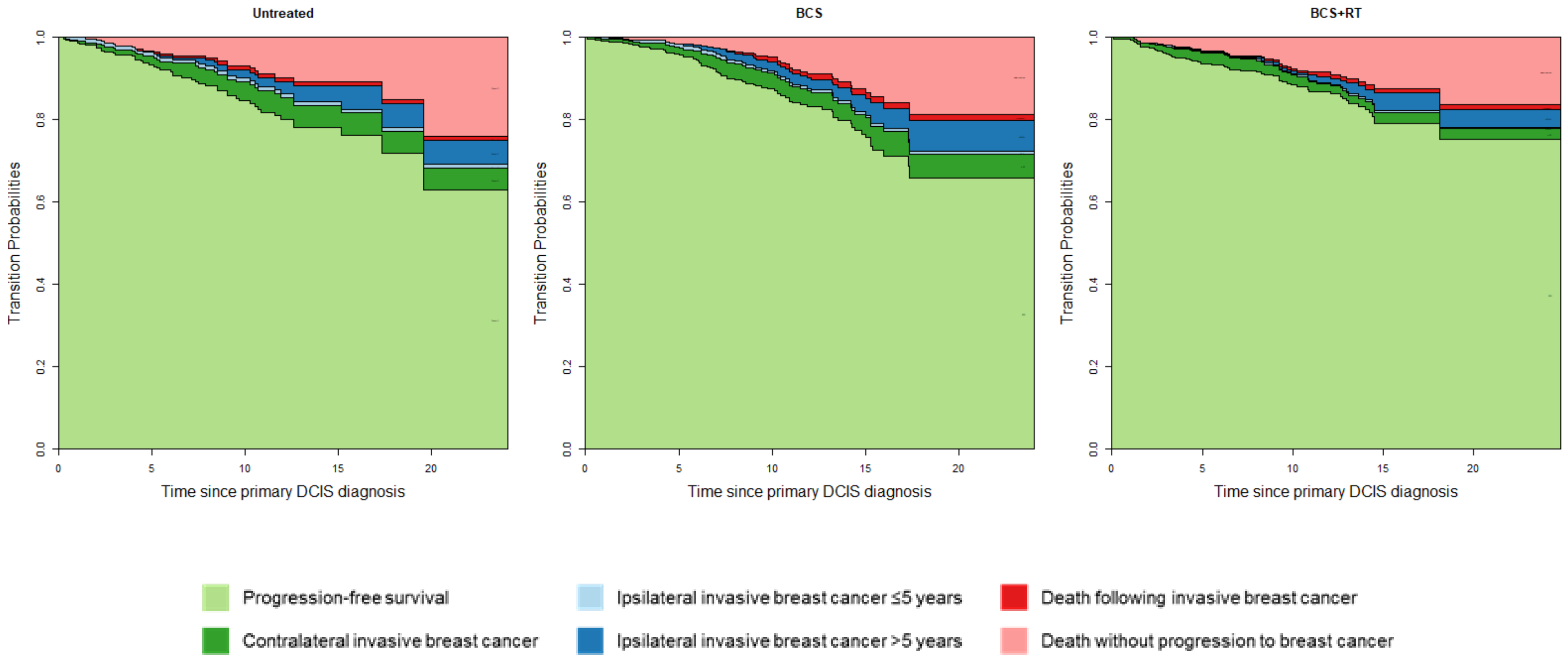

Transition probabilities calculated from the multi-state models are visualized for the different matched 1:2 treatment cohorts within the low-risk DCIS subgroup. These figures coincide with the data in Supplementary Tables 1-8. Each curve represents the instantaneous transition rate (or "progression") to the different possible events of interest over time. The distance between the curves represents an individual's probability of being in a specific health state at a specific time point ("state occupancy probability").
